# P-2297. Evaluation of a Meta-Virus Signature as a Diagnostic Tool to Predict the Severity of Respiratory Viral Infections in Hematopoietic Cell Transplant Recipients

**DOI:** 10.1093/ofid/ofae631.2450

**Published:** 2025-01-29

**Authors:** Tali Shafat, Ronnie LaCombe, Layale Yaghi, Georgios Angelidakis, Amy Spallone, Ella Ariza Heredia, Genevieve S Hanna, Isabelle Ulloa, Rohita Sinha, Pam Morris, Karen Howard-Shipley, Steve Kleiboeker, Fareed Khawaja, Roy F Chemaly

**Affiliations:** The University of Texas MD Anderson Cancer Center, Houston, Texas; Eurofins Viracor, Lenexa, Kansas; UT MD Anderson Cancer CEnter, Houston, Texas; The University of Texas Md Anderson Cancer Center, Houston, Texas; University of Texas MD Anderson Cancer Center, Houston, Texas; The University of Texas MD Anderson Cancer Center, Houston, Texas; Eurofins Viracor, Lenexa, Kansas; Eurofins-Viracor, Kansas City, Missouri; Eurofins Viracor, Lenexa, Kansas; Eurofins Viracor, Lenexa, Kansas; Eurofins Viracor, Lenexa, Kansas; Eurofins Viracor, Lenexa, Kansas; The University of Texas MD Anderson Cancer Center, Houston, Texas; University of Texas MD Anderson Cancer Center, Houston, Texas

## Abstract

**Background:**

Predicting respiratory viral infection (RVI) outcomes may lead to earlier intervention and improved prognosis in immunocompromised hosts. A microarray and RNA-Seq results meta-analysis identified a conserved gene expression host response to RVI of 396 genes called the Meta-Virus Signature (MVS). While the MVS score positively correlates to RVI severity, a 42-gene subset called the Severe-or-Mild (SoM) score distinguishes severity groups with higher accuracy (PMID 33765435). This assay has not been validated for use in immunocompromised patients with RVIs. We aim to determine if SoM scores are associated with the severity of RVIs in hematopoietic cell transplant (HCT) recipients.

**Table 1:**
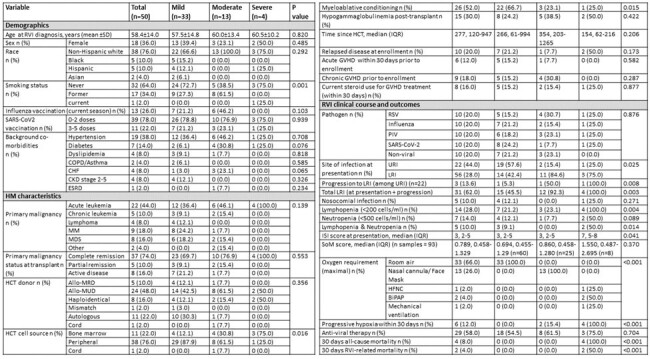
Baseline characteristics and clinical outcomes following respiratory infection (n=50)

Abbreviations: Allo=allogenic, BiPAP= bilevel positive airway pressure, BMI= Body mass index, CKD= chronic kidney disease, COPD= chronic obstructive pulmonary disease, CPAP=continuous positive airway pressure, ESRD= end-stage renal disease, GVHD= graft-versus-host disease, HCT= hematopoietic stem cell transplantation, HFNC= high-flow nasal cannula, HM=hematologic malignancy, ISI= immunodeficiency scoring index, IQR= interquartile range, LRI= lower respiratory tract infection, MRD= matched related donor, MUD= matched unrelated donor, PIV= parainfluenza virus, RSV= respiratory syncytial virus, RVI= respiratory virus infection, SD=standard deviation, SoM= Severe-to-mild, URI= upper respiratory tract infection.

**Methods:**

We prospectively enrolled 40 HCT recipients with different RVIs (respiratory syncytial virus, influenza, parainfluenza, and SARS-CoV-2; 10 for each cohort) and 10 post-transplant patients with respiratory symptoms but negative viral respiratory panel (control cohort). Patients were followed for 30 days. Whole blood was collected in HemaSure-OMICS tubes on Day 0 and Day 5. Expression of the SoM score genes was measured by RT-PCR on the Fluidigm Biomark. Data was normalized to the Delta Ct method using the geometric mean of YWHAZ and UBC Ct values. The SoM score was calculated by taking the sum of the geometric means of previously defined modules 3 and 4 divided by the sum of the geometric means of modules 1 and 2. Infection severity was defined based on 30-day maximal oxygen requirements.**Figure 1.** Median Severe-or-Mild scoring according to 30-day maximal oxygen requirements (among patients with RVI, n=40).

**Figure:**
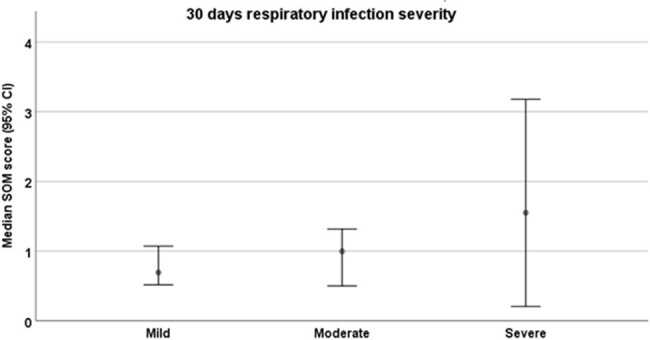
Abbreviations: BiPAP= bilevel positive airway pressure, CPAP= continuous positive airway pressure, IQR= intra quartile range, RVI= respiratory virus infection, SoM= Severe-or-Mild. *Mild infection is defined as no oxygen requirement, moderate infection is defined as the maximal O2 requirement of a nasal cannula or face mask, and severe infection as the need for high flow nasal cannula, BiPAP, CPAP, or mechanical ventilation.

**Results:**

Among the 50 patients enrolled, 33 (66%) had a mild infection, 13 (26%) were moderate, and 4 (8%) experienced severe infections leading to mortality (Table 1). We observed a trend between higher SoM scores and RVI severity, with median SoM scores of 0.694, 0.860, and 1.550 in patients with mild, moderate, and severe infections, respectively (p=0.370) (Figures 1,2). The association between an elevated SoM score and severe infections was stronger for HCT recipients infected with parainfluenza (Figure 2).**Figure 2.** Median Severe-or-Mild scoring and severity of respiratory infection (n=50).

**Figure:**
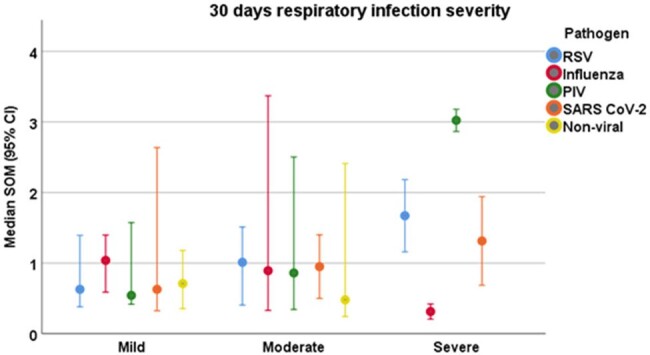
Abbreviations: BiPAP= bilevel positive airway pressure, CPAP= continuous positive airway pressure, PIV= parainfluenza virus, RSV= respiratory syncytial virus, SoM= Severe-or-Mild. *Mild infection is defined as no oxygen requirement, moderate infection is defined as the maximal O2 requirement of a nasal cannula or face mask, and severe infection as the need for high flow nasal cannula, BiPAP, CPAP, or mechanical ventilation.

**Conclusion:**

SOM score was elevated in HCT recipients with severe RVIs, although not statistically significant. Larger prospective cohort studies will be needed to identify clinically relevant applications of gene expression analysis to predict the severity of infection.

**Disclosures:**

Ronnie LaCombe, PhD, Eurofins Viracor: Employee Genevieve S. Hanna, B.S. Biology, Eurofins Viracor: Employee Isabelle Ulloa, n/a, Eurofins-Viracor: Employment Rohita Sinha, PhD, Eurofins Viracor: I am a salaried employee of Eurofins Viracor Pam Morris, M.S., PMP, Eurofins Viracor: Employee Karen Howard-Shipley, M.S., PMP, Eurofins Viracor: Employment Steve Kleiboeker, PhD, Eurofins: employee|Eurofins: Stocks/Bonds (Public Company) Fareed Khawaja, MBBS, Eurofins Viracor: Grant/Research Support|Symbio: Grant/Research Support Roy F. Chemaly, MD/MPH, AiCuris: Advisor/Consultant|AiCuris: Grant/Research Support|Ansun Pharmaceuticals: Advisor/Consultant|Ansun Pharmaceuticals: Grant/Research Support|Astellas: Advisor/Consultant|Eurofins-Viracor: Grant/Research Support|InflaRX: Advisor/Consultant|Janssen: Advisor/Consultant|Karius: Advisor/Consultant|Karius: Grant/Research Support|Merck/MSD: Advisor/Consultant|Merck/MSD: Grant/Research Support|Moderna: Advisor/Consultant|Oxford Immunotec: Advisor/Consultant|Oxford Immunotec: Grant/Research Support|Roche/Genentech: Advisor/Consultant|Roche/Genentech: Grant/Research Support|Shinogi: Advisor/Consultant|Takeda: Advisor/Consultant|Takeda: Grant/Research Support|Tether: Advisor/Consultant

